# Role of Exercise Intensity on Th1/Th2 Immune Modulations During the COVID-19 Pandemic

**DOI:** 10.3389/fimmu.2021.761382

**Published:** 2021-12-22

**Authors:** Rashmi Supriya, Yang Gao, Yaodong Gu, Julien S. Baker

**Affiliations:** ^1^ Faculty of Sports Science, Ningbo University, Zhejiang, China; ^2^ Centre for Health and Exercise Science Research, Department of Sport, Physical Education and Health, Hong Kong Baptist University, Kowloon Tong, Hong Kong SAR, China

**Keywords:** COVID-19, Th1/Th2 ratio, immunomodulation, intermittent hypoxic preconditioning, hypoxia

## Abstract

The COVID-19 pandemic has led to several pioneering scientific discoveries resulting in no effective solutions with the exception of vaccination. Moderate exercise is a significant non-pharmacological strategy, to reduce the infection-related burden of COVID-19, especially in patients who are obese, elderly, and with additional comorbidities. The imbalance of T helper type 1 (Th1) or T helper type 2 (Th2) cells has been well documented among populations who have suffered as a result of the COVID-19 pandemic, and who are at maximum risk of infection and mortality. Moderate and low intensity exercise can benefit persons at risk from the disease and survivors by favorable modulation in Th1/Th2 ratios. Moreover, in COVID-19 patients, mild to moderate intensity aerobic exercise also increases immune system function but high intensity aerobic exercise may have adverse effects on immune responses. In addition, sustained hypoxia in COVID-19 patients has been reported to cause organ failure and cell death. Hypoxic conditions have also been highlighted to be triggered in COVID-19-susceptible individuals and COVID-19 survivors. This suggests that hypoxia inducible factor (HIF 1α) might be an important focus for researchers investigating effective strategies to minimize the effects of the pandemic. Intermittent hypoxic preconditioning (IHP) is a method of exposing subjects to short bouts of moderate hypoxia interspersed with brief periods of normal oxygen concentrations (recovery). This methodology inhibits the production of pro-inflammatory factors, activates HIF-1α to activate target genes, and subsequently leads to a higher production of red blood cells and hemoglobin. This increases angiogenesis and increases oxygen transport capacity. These factors can help alleviate virus induced cardiopulmonary hemodynamic disorders and endothelial dysfunction. Therefore, during the COVID-19 pandemic we propose that populations should engage in low to moderate exercise individually designed, prescribed and specific, that utilizes IHP including pranayama (yoga), swimming and high-altitude hiking exercise. This would be beneficial in affecting HIF-1α to combat the disease and its severity. Therefore, the promotion of certain exercises should be considered by all sections of the population. However, exercise recommendations and prescription for COVID-19 patients should be structured to match individual levels of capability and adaptability.

## Introduction

The immune system is an anatomical defense system that protects an organism from diseases. The immune system is made up of two parts: the innate (general/cell mediated) and the adaptive (specialized/humoral) immune system. The systems perform different tasks individually, together and work in synergy with each other. In the early 20th century, the newly discovered science of immunology was involved in a fundamental debate regarding whether phagocytic cells provided immunity, or if the immunity responsibility was that of humoral antibodies? As the century progressed, the opposing theories coalesced into one paradigm of host immunity: type 1/type 2 immunity. An important biochemical constituent part of the immune system are T helper cells. Initially, all T helper lymphocytes are naive cells (Th0s) that can, following activation, “polarize,” or differentiate, into type 1 (Th1) or type 2 (Th2) lymphocytes (1, 2). The cluster of differentiation 4 (CD4) surface proteins appears on T-helper cells, whereas the cluster of differentiation 8 (CD8) surface protein appears on T-cytotoxic cells. CD4+ T helper cells induce antibody production in B cells and CD8+ T cytotoxic lymphocytes mediate lysis of intracellular pathogen-infected autologous cells. Type 1 immunity is characterized by intense phagocytosis driven by Th1 lymphocytes that secrete interleukin (IL)-2, interferon gamma (IFN-ϒ), and lymphotoxin-a (LT-a). Pro-inflammatory cytokines are primarily related to IFN-ϒ that stimulate phagocytosis (3, 4), the intracellular killing of microbes (5, 6) and the oxidative burst (7, 8). IFN-ϒ also upregulates the expression of class I (9, 10) and class II major histocompatibility complex (MHC) molecules (11, 12) on a variety of cells, thereby stimulating antigen presentation to T cells. Non-leukocytes, such as endothelial cells, are also induced by IFN- ϒ and LT-a (13), fibroblasts (14, 15), and keratinocytes (16), secreting pro-inflammatory cytokines, such as chemotactic cytokines called chemokines (17) and tumor necrosis factor (TNF).

**Figure 1:**
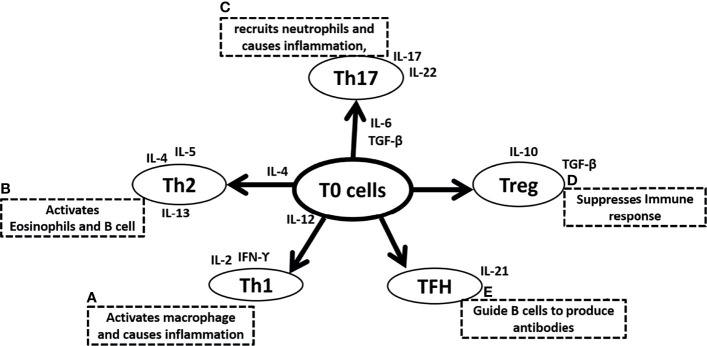
Summary of Naive T (T0) cells differentiation in **(A)** T helper 1 (Th1) cells due to the surrounding microenvironment of IL-12 and further secretes IL-2 and IFN-ϒ and activates macrophage and causes inflammation, **(B)** T helper 2 (Th2) cells due to the surrounding microenvironment of IL-4 and further secretes IL-4, IL-5, IL-13 and activates eosinophil and B cells, **(C)** T helper 17 (Th17) cells due to the surrounding microenvironment of IL-6 and TGF-β and further secretes IL-17, IL-22 to recruit neutrophils and causes inflammation, **(D)** T regulatory (Treg) cells that secretes IL-10, TGF-β and suppresses immune response, **(E)** Follicular helper T (TFH) cells and secretes IL-21, and guide B cells to produce antibodies. IL is interleukins, TGF-β is Transforming growth factor-β and a B cell is B lymphocytes.

Conversely, Type 2 immunity induced by Th2 cells, characterized by highly elevated antibody titers, is characterized by the secretion of interleukin-4, IL-5, IL-9, IL-10, and IL-13. Particularly, IL-4, IL-10, and IL-13 enhance proliferation, activate antibody production, and switch classes of antibodies (18, 19). Hematopoietic cytokine IL-5 is powerful in stimulating eosinophil development in the bone marrow (20–22), as well as activation of eosinophils (23, 24) and basophils (25, 26), whereas Mast cells are stimulated by IL-9, the equivalent hematopoietic factor (27, 28). In addition to their atopic and allergic effects, IL-4, IL-5, IL-9, and IL-13 are strongly implicated in airway inflammation seen in asthma and reactive airway disease (29–33). The T helper cell family include numerous subtypes in addition to Th1 and Th2. Th0, are lymphocytes that are immature or have not polarized during maturation, as well as taking on qualities of both Th1 and Th2 (34, 35). T helper 3 (Th3) and T-regulatory (Treg) are other lymphocyte populations of CD4+ T helper cells. Among mammals, Th3 cells produce transforming growth factor (TGF) β1 that regulates mucosal immunity (36–39). Unlike Th3 cells, Treg cells secrete unusually high levels of IL-10 and lower levels of TGF- β1, which may be implicated in the suppression of immunity in general (40, 41). T follicular helper cells (TFH) guide B cells to produce antibodies and secrete IL-21 (**Figure 1**). It is interesting to note that the imbalance between Th1 and Th2, Treg and Th17 have been well documented among those people who are most susceptible to infection by COVID-19 including the obese, the elderly and individuals with other underlying comorbidities. Patients of COVID-19 and people who have recovered following COVID-19 have also experienced an imbalance between Th1 and Th2, Treg and Th17.

**Figure 2:**
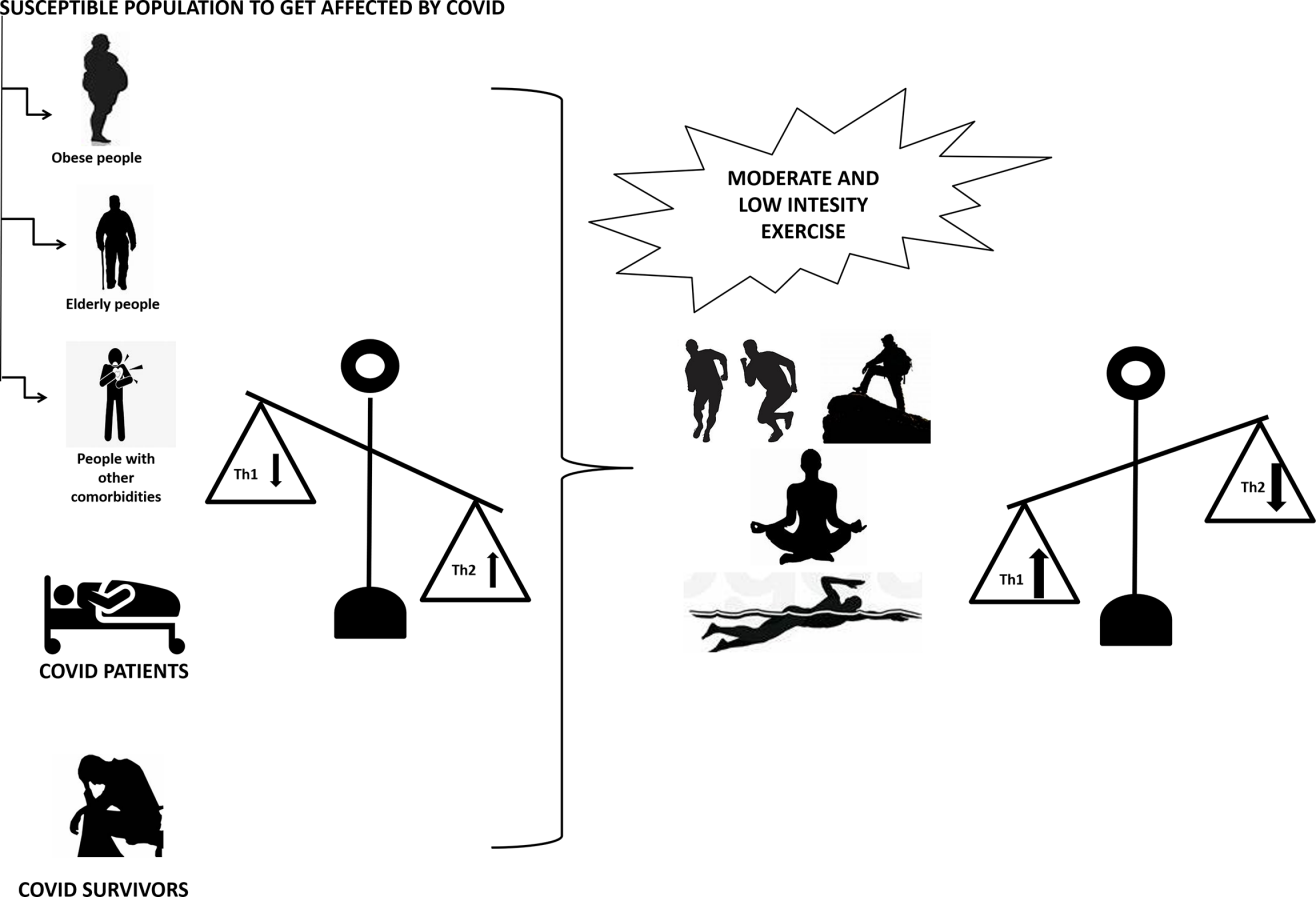
Moderate to low intensity exercise upregulates Th1 and downregulates Th2 in people susceptible to COVID-19 infection (including obese, elderly and people with other comorbidities), COVID-19 patients and COVID-19 survivors with upregulated Th2 and downregulated Th1. Th1 is T helper cells 1 and Th2 is T helper cells 2.

Regular physical exercise promotes immune defense and decreases susceptibility to pathogenic microorganisms, including viruses by modulating the balance of Th1 and Th2 cells, Treg and Th17. A combination of innovative scientific discoveries and clinical observations has revealed several potential drug targets in the midst of the current COVID-19 pandemic. In the meantime, there are currently no effective and safe pharmacotherapies for this novel SARS coronavirus (SARS-CoV-2). Since Th1/Th2 imbalance is linked to SARS-CoV-2 infection, approaches such as non-pharmacological strategies, including moderate exercise, should be considered to reduce the burden of the disease as well as to reduce infection rates (**Figure 2**).

## Populations With Imbalanced Th1/Th2 Are Among the Most Susceptible to Become Infected by COVID 19

### Obese People

COVID-19 courses and mortality are worse when patients are obese, particularly among young (age < 50 years) hospitalized patients (42, 43). In physiochemical terms, the Angiotensin-converting enzyme 2 (ACE2) is known to function as an entry receptor for COVID-19, and obesity may lead to greater levels of ACE2 expression in the lung epithelial cells. This highlights that the more adipocytes there are, the more chances of the virus spreading *via* ACE2 receptors (44). COVID-19 outcomes may be more severe in individuals with high endogenous ACE2 serum concentrations. ACE inhibitors suppress auto reactive Th1 and Th17 cells and promote antigen-specific Treg cells by inhibiting canonical nuclear factor kappa-light-chain-enhancer of activated B cells (NFkβ)-1 transcription factors and activating alternative NFkβ-2 pathways (45). In fact, early clinical results indicate that ACE inhibitor therapy can reduce Th1/Th2 cytokine ratios and inflammatory cytokine levels in patients with chronic heart failure (46). This may also apply to obese populations and warrant further investigation.

Studies conducted prior to the COVID-19 pandemic have demonstrated that intense aerobic exercise acutely raises plasma ACE2 levels in humans (47). The impact of short-term exercise on ACE2 serum concentrations as well as whether and how they will persist over time is unclear. Exercise also induces ACE2 expression in skeletal muscle, but lower levels of this molecule in the bloodstream are believed to impact on COVID-19 pathogenicity. Exercise appears to be a modifiable risk factor, especially for those who are more susceptible to acquiring COVID-19 infections (48). NETs (neutrophil extracellular traps) represent an innate organismic defense mechanism, in which neutrophils release intracellular material which engulf any external agents. The release of NETs is associated with an increase in inflammation and related metabolic diseases, including obesity. In order to treat and prevent obesity, continuous physical training is essential. The link between physical activity, obesity, and NETs has been outlined in a recent bioinformatics analysis. Following ontological analysis, the bioinformatics showed TNF-α to be the leading gene, followed by the regulation of inflammatory response, chemokines, and interleukin-6. The main results of the study indicated that NETs release was regulated by physical training, suggesting a role as a therapy target against NETs and inflammation (49) and modulating the immune system in favor of Th1.

### Elderly Populations

Elderly people with immune compromised states are also associated with poor clinical outcomes from COVID-19 (50). One of the receptor proteins expressed on T cells, a cluster of differentiation 28 (CD28), is required for activating and maintaining T cells. As age progresses, CD28 expression declines and Th1/Th2 are unbalanced, which impairs T-cell-mediated immunity. In a previous study an elderly (age = 61 to 79 years) population of 48 subjects were tested for the effect of moderate exercise, of six months duration on CD28 expression as well as the balance between Th1/Th2 cells (51). The exercise group engaged in moderate-intensity exercise (between 65% and 75% of maximum heart rate maintained in the exercise sessions), including aerobics, light resistance training, and stretches. This study reported that close to 35% of IFN-associated Th1 cell subtypes increased, but Th2 cells associated with IL-4 remained unchanged (52, 53).

The mechanism of immune system improvement due to physical activity is not clearly understood in elderly populations, but one of the contributory factors is the production of free radicals (54–56). Exercise increases oxygen consumption up to 10-fold, resulting in an increase in the concentration of free radicals. The immune system becomes more efficient in combating free radicals in the blood. This is a consequence of increased anti-oxidant enzyme production including superoxide dismutase, catalase and glutathione. There are also increases in cell-mediated immunity, and increased CD4 and CD8 cells (54). Moderate exercise can compensate for decreased sympathetic activity and decreased β adrenergic receptor sensitivity due to aging and may also increase the secretion of catecholamines and stimulate the spleen, proliferation of T cells, CD4 and CD8 in lymph nodes, and lymphatic cells in the thymus (57). β adrenergic receptor stimulation may activate Cyclic adenosine monophosphate and result in lymphocyte production (58, 59) and favorable modulation. With aging, mitochondrial function declines, which increases mortality. According to Santosh Chenoy’s review, mitochondria play a key role in COVID-19 sepsis, specifically the interaction among innate immunity, viral replication, inflammatory state, and HIF-α/Sirtuin pathways. The evidence provided indicates that mitochondria in senescent cells may be dysfunctional, and incapable of maintaining the hypermetabolic demands associated with Covid-19 sepsis. Damage-associated molecular pattern (DAMP) may activate innate immunity through mitochondrial proteins. As oxidative phosphorylation pathways are disrupted, ROS levels increase, which activate the HIF- α/Sirtuin pathway and lead to sepsis. Replication and increased viral load may be enhanced by viral-mitochondrial interaction. In Covid-19 sepsis, hyperoxia and hyperinflammation contribute to increased mortality (60). COVID-19 exhibits immune responses during sepsis. With reduced TH1 responses, and post-septic immunity is characterized by increased TH2-type responses. After sepsis, IFN-γ/IL-4 producing CD4+ T cells appear tilted towards IL-4 (61). Further maintenance of TH1cells after sepsis are negatively affected by becoming more susceptible to activation-induced cell death and apoptosis (62, 63). Specifically, CD4+ T lymphocytes can show decreased proliferation capacity and improper cytokine responses following sepsis, and a defect in the correct regulation of the TH-specific cytokine pathway can negatively influence inflammatory processes post-sepsis. Previous researches suggest that TH1 responses are compromised in post-septic immunity (64).

### Populations With Other Comorbidities

Individuals with comorbidities, such as hypertension, diabetes, coronary heart disease, and chronic obstructive pulmonary disease, are more susceptible to infection and also experience poorer clinical outcomes after COVID-19 (50). Autoimmune diseases are chronic conditions characterized by loss of tolerance to self-antigens. In response to physical activity, Treg1 is elevated, immunoglobulin secretion decreases, and the Th1/Th2 balance shifts. Additionally, physical activity can boost IL-6 levels in the body. Muscle-secreted IL-6 functions as a myokine and inhibits the production of IL-1 β and IL-10, thereby inhibiting inflammation. In most cases of autoimmune diseases such as lupus erythematosus (SLE), rheumatoid arthritis (RA), multiple sclerosis (MS), and inflammatory bowel diseases (IBD), physical activity is safe. In addition, patients less engaged in physical activity have a higher risk of developing RA, MS, IBD and psoriasis. The general trend in patients with autoimmune diseases is that they are less physically active than the general population. RA patients who were physically active had a milder disease course, improved joint mobility and cardiovascular disease (CVD) profile. Patients with MS benefit from physical activity in terms of fatigue reduction, improved mood, and cognitive abilities as well as improved mobility. A higher physical activity level and improved CVD profile have been documented among SLE patients. Exercise can decrease the risk of autonomic neuropathy and cardiovascular disease in people with diabetes mellitus type 1. Physical activity decreases the severity and pain of fibromyalgia and systemic sclerosis symptoms, as well as improving quality of life for patients (65). Researchers studied the relationship between body mass index (BMI) and changes in testosterone/cortisol ratio, serum IL-4/IFN-γ ratio (Th1/Th2 balance) in women with asthma, and to assess changes in cortisol, testosterone, estrogen, and progesterone levels after aerobic exercise training. A total of 21 women, who suffered from mild to moderate asthma and had regular menstrual cycles, participated in this study. The study reported that the exercise group showed a significant rise in Th1/Th2 and a fall in cortisol and BMI, in comparison to the control group. A significant correlation could not be found between cortisol levels, sex hormone levels, BMI, and the increase in Th1/Th2 ratios. It was suggested that in women with asthma, moderate aerobic exercise increased the Th1/Th2 ratio regardless of changes in steroid hormone levels or body mass index (66). An article published in the British Journal of Sports Medicine in 2008 showed that a 12-week program of Tai Chi exercises influenced type 2 diabetics’ Th1/Th2 ratios towards Th1. The study involved 60 patients with type 2 diabetes who were on average 54 years of age. After twelve weeks of tai chi exercises, Th2-inducing IL-4 was lowered and Th1-inducing IL-12 was increased. Whereas, the evidence for the therapeutic effects of higher-intensity exercise in Th2-dominated conditions like asthma or allergies is scarce in human studies, but the potential modulation of inflammation underlying these scenarios may be explored based on Th2-associated cytokines produced by muscle contractions (67).

### COVID-19 Patients Exhibit Imbalanced Th1/Th2

Immune-system imbalances, such as autoimmune issues, allergies, and increased susceptibility to infection, result from an imbalance between Th1 and Th2 cells. The immune response type organized against viral infection is determinant in the prognosis of some infections. An observational prospective study reported Th polarization in acute COVID-19 and its possible relationship with outcomes, was very informative. In a hospital Medical Department, 58 COVID-19 patients were recruited, and 55 patients survived after losses to follow-up. Four Groups were created based on the maximum progression of the disease. Flow cytometry analysis of T-helper cell percentages and phenotypes, as well as Luminex analysis of serum cytokines, were performed upon obtaining the microbiological diagnosis of the disease. In contrast to the reference population, COVID-19 patients had significantly lower %Th1 and %Th17 cells with higher %Th2 cells activated. Senescent Th2 cells were found at higher levels in patients who died compared to those who survived. The senescent Th2 cell percentage (OR: 13.88) was independently associated with death, with a relationship between the total lymphocyte count (OR: 0.15). COVID-19 patients had a profile of serum cytokines that were pro-inflammatory when compared with controls, including IL-2, IL-6, IL-15, and IP-10. Comparing the patients and controls, they also had higher levels of IL-10 and IL-13. IL-15 levels and were significantly higher in patients who died than those who survived. The disease progression groups did not differ significantly from one another. IL-15 and high Th2 responses are associated with a fatal outcome in the study (68). Cytokine IL-15 plays a variety of biological roles in many kinds of cells. As an important player of both innate and adaptive immune responses, it modulates immune cells and causes inflammation and protection from microorganisms (69). Synergistic hypoxia/IL-15 interactions were observed for genes associated with key metabolic and regulatory enzymes based on RT-PCR. In a recent study, it was found that IL-15 stimulates NK cells to switch from glycolysis to anaerobic metabolism (70). The severity of disease was also linked to immune features in another study. It was reported that severe illness was associated with Immunotype 1, characterized by a higher level of CD4 T cell activation, reduced circulating effector CD8 T cells and the presence of B cells in peripheral blood. Moreover, immunotype 2 shows a predominantly effector CD8 T cell subset, less fully activated CD4 T cells, and a more prolific peripheral blood and memory B cell populations. Immunotype 3, in which approximately 20% of COVID-19 patients show minimal activation of lymphocytes, which may be a sign of a lack of antiviral T cell or B cell response. Death occurred in patients with all three immune types, illustrating a complex relationship between immune function and COVID-19 (71).

The SARS-CoV-2 virus binds and infects the cells utilizing ACE-2 as a receptor, which is found in the lungs, kidneys, heart and arteries. ACEs (ACE-1 and ACE-2) act oppositely on the pulmonary endothelium: ACE-2 functions as a vasodepressor, while ACE-1 functions as a vasoconstrictor. In physiological conditions, ACE-1 and ACE-2 are in dynamic equilibrium. Under hypoxemic conditions, such as those associated with COVID-19 infection, HIF-1 upregulates the ACE-1 gene (72). HIF-1α and HIF-2α are both hypoxia-inducible transcription factors that contribute to the hypoxic response and inflammation. Previous studies suggest that the post-translational regulation of both HIFα proteins is very similar. It is unclear how these two isoforms interact functionally. HIF factors control nitric oxide (NO) production in part by controlling macrophage polarization. HIF-1α and HIF-2α are differentially induced by Th1 cytokines during M1 macrophage polarization and by Th2 cytokines during M2 macrophage polarization, respectively. The differential response to polarizing macrophages was most apparent by regulating inducible NO synthase gene expression by HIF-1α and arginase1 gene expression by HIF-2α by different HIF isoforms (73).

As outlined previously, COVID-19 is a condition similar to hypoxia. The effect of exercise on hypoxic conditions has been outlined in many studies. A moderate amount of exercise in normoxia stimulates the immune system whereas a strenuous amount can suppress it. It is less clear how hypoxia influences cytokines during exercise. A previous study was conducted to determine the effects of hypoxic exercise similar to that performed at 4200-meter altitude on cytokine levels. The study demonstrated that under hypoxic conditions, IL-2, the IL-2/IL-4 ratio, and glutamine decreased, but IL-6 and IL-1ra increased. A positive IL-2/IL-4 ratio, an increase in IL-6, IL-1ra, and IL-10/TNF-α were reported in normoxia. In terms of cortisol or glucose, there was no difference. The study concluded that IL-2, IL-4 and TNF-α cytokines were changed by moderate exercise in hypoxia, suggesting a Th2-like response following 1 hour of rest (74). It has also been proposed that stress hormones are responsible for mediating the exercise- and hypoxia-induced changes in leukocyte subpopulations. Noradrenaline and adrenaline both contribute to the acute effects that adrenaline has on lymphocyte subpopulations, lymphokine-activated killer (LAK) cells and natural killer cells. LAK cells react to these cytokines, particularly IL-2, by lysing tumor cells that are already resistant to natural killer (NK) cells (75). Following prolonged exercise, growth hormone stimulates neutrophils quickly, while cortisol exerts its effects much more slowly, and contributes to maintaining lymphopenia and neutrocytosis. The mechanisms underlying both exercise and hypoxia differ markedly in terms of their impact on plasma IL-6 levels. The enormous increase in plasma IL-6 during strenuous exercise contributes only in a minor way to the increased level of adrenaline. In contrast, the prolonged increase in IL-6 during chronic hypoxia may be due to several factors, including hormonal changes. Although exercise is performed at the same relative workload in normoxia and hypoxia, the relative intensity of the exercise increases during hypoxia, which may explain why hypoxia induced changes in leukocyte subpopulations and plasma-IL-6 are more pronounced when exercising during hypoxic conditions. Increasing exercise intensity may partly be responsible for the more pronounced immune responses induced by exercise under hypoxic conditions. Several oxygen signaling molecules, including HIF-1α, seem to play some role in hypoxia-induced alterations (76), and the same factors may be involved during exercise at altitudes or during hypoxia (77). However, HIF-2α has not been studied in hypoxic exercise conditions.

COVID-19 is a relatively new disease, so there are still no definitive scientific guidelines on how patients should mobilize and exercise in the early stages of the disease. The most challenging aspect of exercise prescription for pulmonary patients, according to the American Association for Cardiovascular and Pulmonary Rehabilitation, is determining the appropriate intensity of exercise to ensure exercise does not cause adverse physiological effects, while promoting positive effects (78). Determining an ideal exercise intensity can be even more challenging when dealing with a new disease lacking in scientific baseline information. Some studies suggest the value of exercise as a rehabilitation tool for pneumonia patients with other etiologies (79, 80). Low intensity exercises are most often recommended for mild patients according to exercise prescription guidelines (81–84). Patients who develop severe COVID-19 and require mechanical ventilation and stay in hospital for a long time are at risk for ICU-acquired weakness. During these periods, mobilization can help improve the patient’s cognitive, respiratory, and functional conditions, allowing the patient to be discharged earlier (85). It has been noted that exercising can enhance the immune system in healthy, asymptomatic individuals and that exercise engagement is appropriate for the current epidemic of viral respiratory illnesses (86–88). Moderate exercise has been shown to have the following immunological benefits: increases neutrophils and NK cells counts, as well as enhancing stress hormone levels, which in turn reduce inflammation (89). Furthermore, aerobic exercises should be avoided during high fever in order to prevent a decrease in immunity (90).

### COVID-19 Survivors Exhibit Imbalanced Th1/Th2

In the later stages of recovery, T1, T2, and Th17 cell percentages in patients were lower than those of healthy controls, whereas they increased when the disease was in remission. A significant reduction in IL-1α, IL-1β, IL-6, TNF-α, and IL-10 levels was reported in the late recovery stage. In the process of recovery, the level of TGF-β1 did not significantly change. TGF-β may be produced by Treg cells. In contrast, TGF-β1 production in T cells triggers Th1 and Th2 responses (91). When disease recovery occurs, the immune system improves and regulates immune responses and reduces the severity of the disease (92). COVID-19 survivors generally suffer from chronic fatigue, depression, stress, anxiety and psychological problems. People with chronic fatigue syndrome (CFS) suffer from severe fatigue that cannot be relieved by rest. It usually occurs after an infection or a stressful event. Activated lymphocytes in patients with CFS exhibit poor immune cell function and result in predominantly Th2-type cytokine responses. Th2 cells and their cytokines (IL-4, VIL-5, and IL-10) are the cellular factories for immunoglobulins that produce a Th2-type response and are responsible for producing these cytokines. Pathologies, such as autoimmunity, are characterized by an excess formation of immunoglobulins and, therefore, a Th2-type response predominates. As a result of COVID-19 recovery, patients usually suffer from stress, anxiety, and depression (93). Studies have demonstrated that anxiety and depression impair immunity (94–96). Moreover, stress plays a crucial role in morbidity and mortality rates associated with immune-mediated diseases (94). Psychological problems can affect immunity by altering the balance of immune cells, for example altering the balance of Th-1/Th-2 cells. During psychological stress and psychiatric disorders, the balance between cytokine Th1 and cytokine Th2 plays a critical role in modulating brain cellular responses. The plasma levels of IFN-ϒ, IL-4, and TGF-β1 were measured during admission as well as 8 weeks after treatment with antidepressants. Depressed patients had significantly higher plasma IFN-ϒ/IL-4 ratios and immune reactivity to both IFN-ϒ and IL-4 than controls. The IFN-ϒ/TGF-β1 ratio has also been reported to be higher in depressed COVID-19 patients, and TGF-β1 levels showed a significant negative correlation with depression on the Hamilton depression rating scale. An imbalance in Th1 and Th2 cytokines was observed in subpopulations of depressed patients. TGF-β1 appeared to play an important role in the pathophysiology of depression in this population (97). Academic exam stress and caregiver’s stress can shift the balance of Th1/Th2 cytokines to Th2, thereby promoting an inflammatory response in humans. In susceptible individuals, psychological stress can trigger the activity of indoleamine 2,3 dioxygenase and cause disorders related to serotonin depletion, such as depression. Psychological stress can lead to the production of cytokines which can lead to an increase in the risk of developing certain diseases, such as cardio-vascular disease and autoimmune disease. Importantly, psychological stress may cause the production of neurodegenerative cytokines in the brain (98). Stress increases Th1 cytokine production and its receptor expression. Acute stress results in increased catecholamine-mediated signal pathways to the nervous and immune systems, but not the glucocorticoid receptor, which is consistent with the hypothesis that the central nervous system and immune system play a fundamental role in acute stress-mediated immune disorders (99). In addition to increasing serum corticosteroids and catecholamines (CAs), stress might also cause a decrease in immunity (94). Corticosteroids are a class of steroid hormones, produced in the adrenal cortex of vertebrates. Glucocorticoids and mineralocorticoids are two of the two main types of corticosteroids, and they trigger stress response, immune response, regulation of inflammation, carbohydrate metabolism, protein breakdown, electrolyte balance, and behavioral response. The effects of corticosteroid therapy on the lungs appear to be related to the normal balance between Th1, Th2 cytokines and immunoglobulins (100). A CAs is a hormone produced by the adrenal glands, which are located on top of the kidneys. CAs include dopamine; norepinephrine; and epinephrine. When individuals are stressed physically or emotionally, our adrenal glands secrete CAs into our bloodstream. Cellular apoptosis and lymphocyte proliferation are regulated by the CAs produced by lymphocytes. A study explored how lymphocytes-derived CAs affects T cell differentiation and function. Lymphocytes synthesize and secrete CAs that regulate differentiation and function of Th cells, enabling the shift of Th1/Th2 balance toward Th2 polarization (101).

In many of the CFS therapies mentioned, the Th2-type response seen at baseline is decreased, allowing Th1 responses to predominate. It is generally reported that cancer survivors suffer from CFS. In a recent study, a 16-week Tai Chi exercise intervention was assessed for its effects on postoperative non-small cell lung cancer survivors. They reported that 16 weeks of Tai chi reduced the natural recovery process of the decreased T1/T2 ratio. A role for Tai Chi in treating the imbalance between cellular and humoral immunity may be possible (102). The association between exercise duration and mood variation has been demonstrated to be non-linear, and performing 10- to 30-minutes of aerobic exercise is enough to boost mood (103, 104). When the sympathetic system is stimulated by moderate-to-vigorous physical activity, CA’s such as adrenaline, noradrenaline, and dopamine are released which play a role in the metabolic processes and immune system. Depending on the intensity and duration of PE, there is a significant role for CAs in lipid and carbohydrate metabolism, immunity, and for generating reactive oxygen species (105). Increasing the aerobic capacity can significantly improve mood. Aerobic exercise may be responsible for this due to the effect on stress hormones, including corticosteroids and CAs hormones, which can help rebalance TH1/TH-2 relationship (106). In contrast, high-intensity workouts and long-term workouts raise Cortisol while low-intensity exercises and relaxing activities lower concentrations. Studies have shown that elevated levels of Cortisol can shift Th1 functions towards Th2 functions (107). It is also possible that high-intensity exercise will increase muscle-derived IL-6 (which has a different behavior from TNF-α-associated IL-6) and associated IL-10, causing a more Th2-dominant state in the body. IL-6, which is derived from muscle tissue, and IL-10 have anti-inflammatory action and influence the immune response of the body (108). Regular and sporadic physical activity apart from low-intensity exercise does not seem to significantly impact cell-mediated immunity (52, 109). Individuals with a poor physical condition or with underlying health conditions should avoid too much exercise and engage in low-intensity exercises such as yoga, which can help relieve stress. Despite yoga’s less restrictive breathing and physical strain, it is effective for the humoral defense system (110, 111). Yoga’s ability to enhance cellular immunity remains inconclusive. Through TGF β1, antidepressant treatment affects the Th1/Th2 balance (97). It is thought that exercise intensity contributes to Th1/Th2 imbalance by affecting the dynamics of cytokines and hormones. Walks, yoga, and tai chi elicit the Th1 response when performed at a low intensity (52, 53, 112), while increasing intensity and duration of workouts push Th2 to the other side of the equation (113–115). In numerous aspects of human health, regular moderate physical activity and the negative effects of severe exercise and/or overtraining have been documented, including the reduction of cardiovascular disease and certain types of cancer.

### Is HIF-1α the Key Target for a Cure in the COVID-19 Era?

Cytokine storms triggered by SARS-CoV-2 is an essential characteristic of COVID-19 and a critical determinant of COVID-19 prognosis (116, 117). In a study published in 2021, researchers found that SARS-CoV-2 ORF3a and host HIF-1α play key roles in viral infection and pro-inflammatory responses. ORF3a (formerly called X1 or u264) (118) has been identified in several sarboviruses that cause SARS, including SARS-CoV (118, 119) and SARS-CoV-2 (120, 121). As the ORF3a of SARS-CoV-2 induces mitochondrial damage and mitochondrial ROS production, it promotes the expression of HIF-1α, which in turn facilitates SARS-CoV-2 infection and cytokine production. Moreover, HIF-1α also promotes infection by other viruses. As a collective unit, ORF3a increases HIF-1 α expression during SARS-CoV-2 infection, which in turn aggravates viral infection and inflammatory responses. As a result, HIF-1 α promotes SARS-CoV-2 infection and induces pro-inflammatory responses to COVID-19 (122). According to RNA sequencing, COVID-19 patients have dysregulated HIF-1α signaling, immune response, and metabolism pathways. In elderly patients, HIF-1 α production, inflammatory responses, and high mortality rates have been shown in clinical studies. Infected cells and patients are elicited to produce HIF-1α and pro-inflammatory cytokines. HIF-1α is a vital metabolic sensor involved in hypoxic responses (123). Sustained hypoxia is a primary pathophysiologic feature and main cause of mortality in patients with severe COVID-19 and it accompanies all the stages of the disease (124). We must also be aware that even in obesity (125), elderly (126), with other comorbidities (127, 128) and even COVID-19 survivors (129), HIF-1α has been reported to be upregulated compared to normal populations. HIF has been the dominant player reported to upregulate ACE in pulmonary smooth muscle cells and circulation (130) (reported high in obese subjects). By Stat3-dependent stimulation of Th17, HIF-1 mediates Foxp3 degradation and negatively regulates Treg development, HIF-1 attracts neutrophils and causes inflammation such as autoimmunity (131).

**Figure 3:**
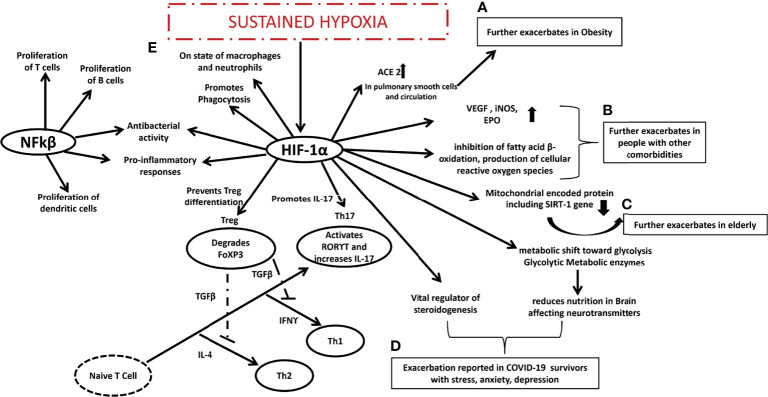
Expected mechanism proposed in COVID-19 era. **(A)** HIF-1 α is already exacerbated in obese people and it might further exacerbates ACE in pulmonary smooth cells and circulation making the obese more susceptible to COVID-19 infection, **(B)** HIF-1 α is already exacerbated in people with other comorbidities and it might further exacerbates VEGF, iNOS, EPO, inhibits fatty acid B oxidation, produces cellular reactive oxygen species making the people with other comorbidities more susceptible to COVID-19 infection, **(C)** HIF-1 α is already exacerbated in elderly people it further reduces mitochondrial encoded protein including SIRT1 gene making the elderly people more susceptible to COVID-19 infection, **(D)** HIF-1 α is vital regulator of steroidenesis and it leads to shift towards glycolysis and reduces nutrition in brain affecting neurotransmitters that may be the reason behind stress depression and anxiety among COVID-19 survivors, interaction between **(E)** NFkβ and HIF-1α might be the reason behind sustained hypoxia in COVID-19 patients promoting Th17, Th1, Th2 and suppressing Treg cells. HIF-1 α is hypoxia induced factor 1 alpha, Th is T helper cells, ACE is Angiotensin-converting enzyme, VEGF is Vascular endothelial growth factor, iNOS is inducible nitric oxide synthase, EPO is Erythropoietin, SIRT1 is Sirtuin 1, NFkβ is nuclear factor kappa-light-chain-enhancer of activated B cells, RORϒT is Retineic-acid-receptor-related orphan nuclear receptor gamma, IFNϒ is interferon gamma, FoXP3 is forkhead box P3.

A variety of pathological conditions are associated with hypoxia and inflammation, especially inflammatory disorders. HIFs are mainly activated by hypoxia, but NFkβ transcription factors are primarily activated by inflammation (132). HIF-1α decreases mitochondrial encoded proteins, which leads to decreased SIRT1 gene expression, increasing their susceptibility to infections from COVID-19 (126). Additionally, HIF-1α and β heterodimers promote cell survival, glycolysis, and angiogenesis, and have been reported dysfunctional in people with type 2 diabetes (133) and cancer (134) making people with other comorbidities more susceptible to COVID-19 infection. In addition to the obese, and the elderly, and those with comorbidities who are more susceptible, the HIF-1α gene has been reported to be activated in patients who have survived COVID-19 infections (135). Research has shown that HIF-1α increases the glycolytic metabolic enzyme pyruvate dehydrogenase kinase 1(PDK1) which reduces nutrition in the brain in the long term, affecting neurotransmitters. The modulation of HIF-1α in adrenocortical cells in mice suggests that it is a key regulator of steroidogenesis (136). Since the HIF-1α gene regulates all three types of populations that struggle the most during COVID-19 infection, it might be the core regulator administering different roles in each type of population (**Figure 3**).

### Can Exercise Target the Key Player HIF-1α During COVID-19 Infection?

**Figure 4:**
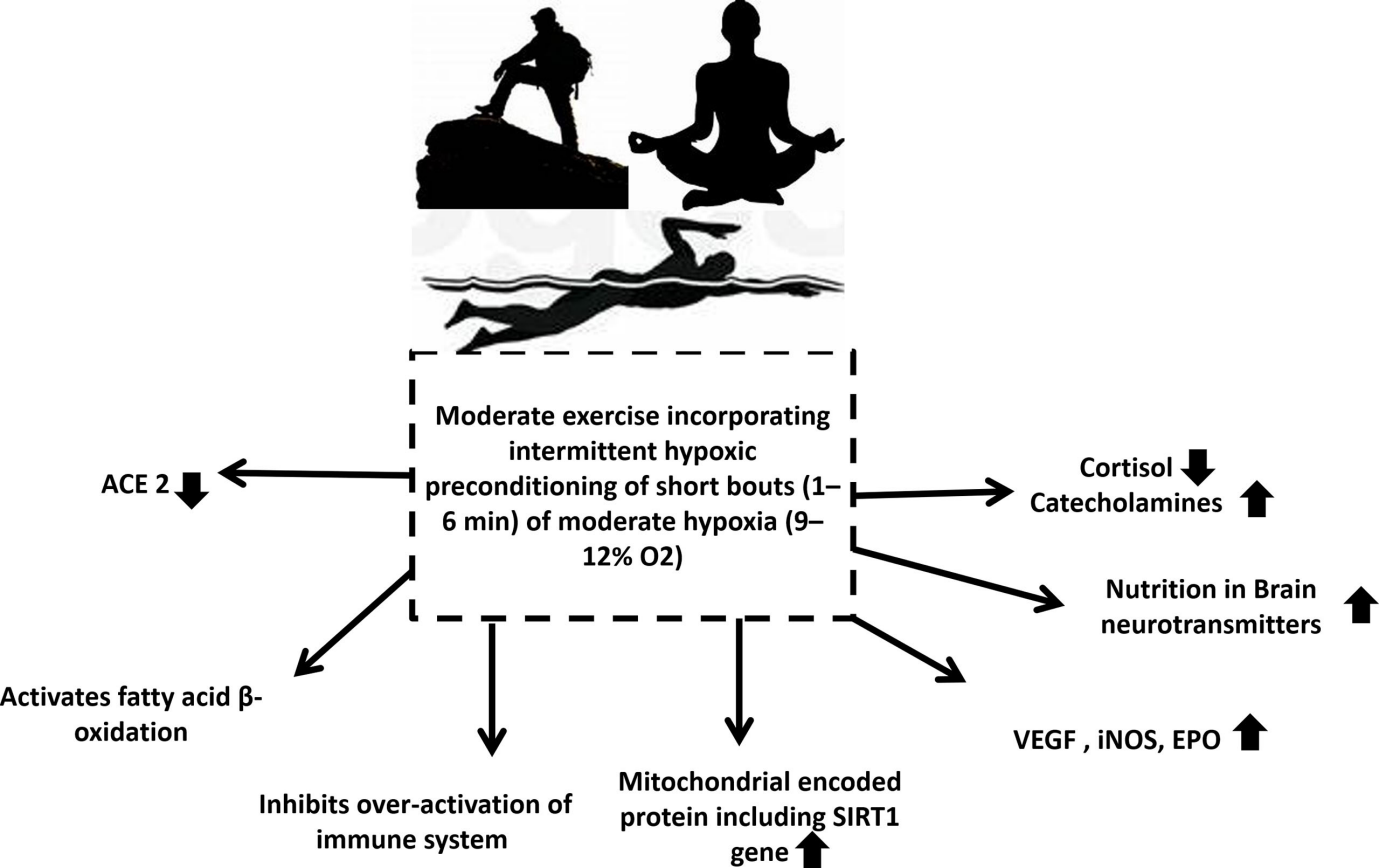
Exercise that involves intermittent hypoxic preconditioning might be the solution for all populations who are at highest risk of infection and mortalities during the COVID-19 pandemic. It decreases cortisol levels, increases catecholamine levels, increases nutrition in brain modulating neurotransmitters, increases VEGF, iNOS, EPO, increases mitochondrial encoded protein including SIRT1 gene, inhibits over activated immune system, activates fatty acid β oxidation and reduces ACE2. ACE is Angiotensin-converting enzyme, VEGF is vascular endothelial growth factor, iNOS is inducible nitric oxide synthase, EPO is Erythropoietin, SIRT1 is Sirtuin 1.

Intermittent hypoxic preconditioning (IHP) is a method of exposing subjects to short (1 to 6 minutes) bouts of moderate hypoxia (9–12% Oxygen) interspersed with brief periods of normal oxygen (137). Possibly due to oxidative stress, intermittent hypoxia can result in a delayed response of HIF-1α, activating NFkβ inflammation (126). By applying IHP to patients, it can inhibit the production of proinflammatory factors, activate HIF-1α to activate target genes, and subsequently lead to a higher production of red blood cells and hemoglobin, while increasing angiogenesis to increase oxygen transport capacity. Additionally, activated HIF-1α may activate the peroxisome proliferator-activated receptor-gamma coactivator (PGC-1)-SIRT1/adenosine monophosphate-activated protein kinase (AMPK) pathway and inhibit the endothelin 1(ET-1) pathway. These factors can help reverse the virus induced cardiopulmonary hemodynamic disorder and endothelial dysfunction (135). A study suggests combined intermittent exercise with hypoxia enhanced glucose disposal and improved insulin resistance post-exercise was beneficial in the treatment of type 2 diabetes (138). Research has also shown that IHP has therapeutic benefits in other neuropathology’s such as alcohol withdrawal stress and Alzheimer’s disease (139) (**Figure 4**). In addition to relaxing airways and blood vessels and improving myocardial contractility, IHP can trigger the body’s endogenous protective mechanism. In addition, it increases cardiopulmonary endurance, reduces heart infarctions, increases blood vessel density, and coordinates the delivery of oxygenated blood. Thus, it has significant defense and protective effects against subsequent prolonged or severe ischemia’s and hypoxia (140). Apart from this, it also improves respiratory muscle function and relieves dyspnea, alleviates depression and anxiety associated with disease, and enhances upper and lower limb muscular function (141). It has been proven that IHP stimulates cellular defenses against oxidative stress and inflammation by improving rat immune systems (142), however such studies are lacking in humans. Secondly, IHP is also effective for reducing cardiopulmonary damage (143). Furthermore, studies revealed that IHP can not only activate HIF-1α, AMPK/SIRTl/PGC-1α but also reduce the protein and mRNA levels of ACE2 thereby inhibiting the ability of SARS-CoV-2 to enter host cells by reducing the number of receptors, improving endothelial dysfunction, promoting cardiovascular hemodynamic and inhibiting excessive inflammation and immune response. Changes like these will benefit individuals recovering from heart and lung injuries or dyspnea (130, 144–146). Further to this, inhibition of ACE2 by HIF-1α could provide a novel method of treating COVID-19 with IHP (**Figure 4**). Considering that there are no precise medical methods for treating or preventing COVID-19, we propose that the many benefits of IHP, has the potential to improve the immunity of individuals susceptible to the disease, accelerate patient recovery, and reduce the risk of positive rejuvenation after discharge. Kumbhaka (“breath retention”), a type of pranayama and swimming have both been demonstrated to induce a state of intermittent hypoxia (147, 148). Many exercise conditions may lead to intermittent hypoxia followed by periods of normoxia or less hypoxic conditions including high altitude mountaineering (149). These conditions need to be explored in detail as innovative and exciting developments in the treatment of COVID-19.

## Conclusion

In conclusion, moderate and low intensity exercise has beneficial effects for individuals who are at risk from COVID-19 as well as those who have recovered physically by increasing Th1/Th2 ratios. Also, the performance of low to moderate intensity aerobic exercises is beneficial in increasing the function of the immune system in patients with COVID-19. However, individuals who are immunosuppressed should avoid performing high intensity aerobic exercise, as this may have adverse effects on their health (150). The effects of aerobic exercise on decreasing immunity are also detrimental in populations with high fevers (90, 151) and therefore, exercise should be avoided in these individuals. There is no consensus of opinion concerning the amount and intensity of exercise that should be recommended for COVID-19 patients, as exercise may result in decreasing Th1/Th2 ratios. COVID-19 patients have shown that the severity of the disease is directly proportional to the decrease in the ratios of Th1/Th2. Adaptability should be considered when recommending exercise for COVID-19 patients. Additionally, we recommend that individuals of all ages, including people with COVID-19 should select exercises that include IHP. IHP involves increasing blood oxygen delivery and promoting tissue oxygenation responses resulting in a reduced susceptibility to COVID-19 infection. This type of activity reverses endothelial dysfunction, improves cardiovascular health, inhibits excessive inflammation, and improves mental health in COVID-19 survivors. Additionally, IHP has the advantage of being extremely safe, easy to perform, and without side effects (138). IHP seems a viable alternative to pharmacological interventions for COVID-19 patients. Providing that the exercise intensity is individually prescribed, specific and monitored. Research suggests that this type of activity may have profound effects for COVID-19 sufferers and may provide substantial relief pre and post infection.

## Author Contributions

RS wrote the paper. JB and RS designed the study. JB, YG, and YDG contributed to discussion and editing. All authors contributed to the article and approved the submitted version.

## Funding

Key Project of the National Social Science Foundation of China (19ZDA352), NSFC-RSE Joint Project (81911530253), Zhejiang Province Science Fund for Distinguished Young Scholars (R22A021199) and K. C. Wong Magna Fund in Ningbo University.

## Conflict of Interest

The authors declare that the research was conducted in the absence of any commercial or financial relationships that could be construed as a potential conflict of interest.

## Publisher’s Note

All claims expressed in this article are solely those of the authors and do not necessarily represent those of their affiliated organizations, or those of the publisher, the editors and the reviewers. Any product that may be evaluated in this article, or claim that may be made by its manufacturer, is not guaranteed or endorsed by the publisher.
